# Muscularity-Oriented Eating as a Predictor of Functional Impairment in Medical Students

**DOI:** 10.1192/j.eurpsy.2026.12025

**Published:** 2026-07-31

**Authors:** N. Usta Sağlam, O. Demirci, I. Yıldırım, E. Serraç, A. F. Çelik, B. Uçar Bostan, M. M. Kırpınar, C. Aksoy Poyraz

**Affiliations:** 1 Psychiatry, Istanbul University-Cerrahpasa , Cerrahpasa Faculty of Medicine, Istanbul; 2 Psychiatry, Kafkas University, Faculty of Medicine, Kars; 3 Istanbul University-Cerrahpasa , Cerrahpasa Faculty of Medicine; 4 Psychiatry, Bakırköy Research and Training Hospital for Psychiatric and Neurological Diseases, Istanbul, Türkiye

## Abstract

**Introduction:**

Eating disorders (EDs) are closely linked to idealized body image, which has increasingly shifted toward a lean and muscular physique. Muscularity-oriented disordered eating (MODE) refers to dietary behaviors aimed at increasing lean muscle mass. Although MODE has traditionally been associated with men, growing evidence suggests rising engagement among women. EDs are known to impair functioning significantly, yet the specific contribution of MODE remains unclear, especially in groups considered at elevated risk for disordered eating, such as medical students.

**Objectives:**

This study examined the independent role of MODE in functional impairment and depressive symptoms among men and women medical students.

**Methods:**

Of the 2,197 students contacted, 471 participated (62.4% women). Participants completed the Muscularity-Oriented Eating Test, the Eating Disorder Examination Questionnaire-Short Form, the Patient Health Questionnaire-9, the Clinical Impairment Assessment, and the Rosenberg Self-Esteem Scale to assess MODE and ED behaviours, depressive symptoms, functional impairment, and self-esteem, respectively. Hierarchical multiple regressions were conducted separately for women and men.

**Results:**

A history of an eating disorder was reported by 5.3% of participants, more common among women (7.8% vs. 1.1%, p = .002). Women also reported lower self-esteem, higher depressive symptoms, and greater functional impairment (all p < .05), while men reported higher supplement use. Regression models explained 71% of variance in functional impairment in women and 56% in men (all p < .001). MODE remained a significant independent predictor, accounting for 9% unique variance in women (β = .40, p < .001) and 5% in men (β = .36, p < .001), beyond thinness-oriented eating and depressive symptoms. In contrast, models for depressive symptoms explained 37% of variance in women and 34% in men. Low self-esteem was the strongest predictor (β = .41 in women; β = .54 in men; both p < .001), while MODE contributed minimally and nonsignificantly (p > .05).

**Conclusions:**

Our findings suggest that MODE represents a distinct dimension of eating pathology, exerting a strong impact on functional outcomes in both women and men. Recognizing and evaluating MODE may therefore be clinically valuable, and interventions tailored to this pattern of eating behavior could help reduce associated psychosocial difficulties. Furthermore, our results highlight the presence of disordered eating behaviors among medical students, underscoring the importance of addressing this at-risk group in both clinical practice and future research.

**Disclosure of Interest:**

None Declared

